# HOXB13 interactome in prostate cancer cells: biochemical and functional interactions between the transcription factors HOXB13 and TBX3

**DOI:** 10.18699/vjgb-25-82

**Published:** 2025-10

**Authors:** М.M. Erokhin, N.Y. Kozelchuk, R.H. Ziganshin, V.V. Tatarskiy, D.A. Chetverina

**Affiliations:** Institute of Gene Biology of the Russian Academy of Sciences, Moscow, Russia; Institute of Gene Biology of the Russian Academy of Sciences, Moscow, Russia; Shemyakin and Ovchinnikov Institute of Bioorganic Chemistry of the Russian Academy of Sciences, Moscow, Russia; Institute of Gene Biology of the Russian Academy of Sciences, Moscow, Russia; Institute of Gene Biology of the Russian Academy of Sciences, Moscow, Russia

**Keywords:** prostate cancer, transcription factors, regulation of transcription, HOXB13, TBX3, TBX2, ZFHX4, ZFHX3, RUNX1, NFAT5, рак простаты, транскрипционные факторы, регуляция транскрипции, HOXB13, TBX3, TBX2, ZFHX4, ZFHX3, RUNX1, NFAT5

## Abstract

Transcription factors represent one of the major groups of proteins, whose suppression leads to tumor growth arrest. Different types of cancer express a specific set of transcription factors that create and maintain unique patterns of gene expression. In prostate cancer cells, one of the key transcriptional regulators is the HOXB13 (Homeobox B13) protein. HOXB13 is known to be an important regulator of embryonic development and terminal cell differentiation. HOXB13 regulates the transcription of many genes in normal and transformed prostate cells and is also capable of acting as a pioneer factor that opens chromatin in the regulatory regions of genes. However, little is known about the protein partners and functions of HOXB13 in prostate cells. In the present study, we searched for protein partners of HOXB13 by immunoaffinity purification followed by high-throughput mass spectrometric analysis (IP/LC-MS) using the PC-3 prostate cancer cell line as a model. The main partners of HOXB13 were found to be transcription factors with different types of DNA-binding domains, including the TBX3, TBX2, ZFHX4, ZFHX3, RUNX1, NFAT5 proteins. Using the DepMap resource, we have shown that one of the identified partners, the TBX3 protein is as critical for the growth and proliferation of prostate cancer cell lines in vitro as HOXB13. Analysis of individual prostate cancer cell lines revealed that knockout of both genes, HOXB13 and TBX3, leads to the death of the same lines: VCaP, LNCaP (clone FGC), PC-3 and 22Rv1. Thus, HOXB13 and TBX3 can be considered together as potential targets for the development of specific inhibitors that suppress prostate cancer cell growth.

## Abstract (ru)

Транскрипционные факторы относятся к одной из главных групп белков, подавление активности которых приводит к остановке роста опухолей. В различных типах рака экспрессируется определенный набор транскрипционных факторов, которые создают и поддерживают специфические паттерны экспрессии генов. В клетках рака простаты ключевым транскрипционным регулятором является белок HOXB13 (Homeobox B13). Известно, что HOXB13 – важный регулятор эмбрионального развития и терминальной клеточной дифференцировки. Он регулирует транскрипцию многих генов в нормальных и трансформированных клетках простаты, а также способен действовать как пионерный фактор, который открывает хроматин в регуляторных областях генов. Однако данных о белковых партнерах и функциях HOXB13 в клетках рака простаты очень мало. В настоящей работе мы провели поиск белковых партнеров HOXB13 методом иммуноаффинной очистки с последующим высокопроизводительным масс-спектрометрическим анализом (IP/LC-MS), используя в качестве модели клеточную линию рака простаты PC-3. Было обнаружено, что основными партнерами HOXB13 являются транскрипционные факторы с разными типами ДНК-связывающих доменов, в том числе белки TBX3, TBX2, ZFHX4, ZFHX3, RUNX1, NFAT5. С помощью ресурса DepMap мы показали, что один из установленных партнеров, белок TBX3, как и HOXB13, критически важен для роста и пролиферации клеточных линий рака простаты in vitro. Анализ отдельных клеточных линий рака простаты выявил, что нокаут обоих генов, HOXB13 и TBX3, приводит к гибели одних и тех же линий: VCaP, LNCaP (clone FGC), PC-3 и 22Rv1. Таким образом, HOXB13 и TBX3 могут совместно рассматриваться как потенциальные мишени для создания специфических ингибиторов, подавляющих рост клеток рака простаты.

## Introduction

Prostate cancer is the most commonly diagnosed cancer among men and is one of the leading causes of male cancer mortality (Siegel et al., 2023). Currently, the most common way to target prostate cancer cells chemically is to block the androgen receptor, AR. However, in most cases, tumor cells become resistant to this type of therapy over time, resulting in the development of “castration-resistant prostate cancer” (CRPC) (Crona, Whang, 2017). In this regard, it is important to identify targets for developing new inhibitors of prostate cancer tumor progression.

The transcription factor HOXB13 is a potential target for prostate cancer therapy. This protein is encoded by one of 39 homeobox genes, which contain a DNA-binding HOX domain (also called a homeobox). These genes control transcriptional cascades in various tissues under normal and pathological conditions (Feng et al., 2021; Hubert, Wellik, 2023). HOXB13 was found to be a pioneer factor, with its binding sites often overlapping with those of FOXA1, GATA2 and other DNA-binding proteins in cell lines originating from prostate tissue (Hankey et al., 2020; Pomerantz et al., 2020). HOXB13 expression levels are elevated in approximately 85 % of prostate adenocarcinoma cases, and this correlates with resistance to AR-targeted therapy, as well as with metastasis and tumor recurrence during treatment (Zabalza et al., 2015; Yao et al., 2019; Weiner et al., 2021). HOXB13 mutations in tumor cells have also been shown to be associated with a poor prognosis for prostate cancer patients (Ewing et al., 2012; Cai et al., 2015; Adashek et al., 2020).

Despite the important role of HOXB13 in prostate cancer cell proliferation, its biochemical and functional properties are poorly understood. In the present study, we analyzed the HOXB13 protein interactome in the PC-3 prostate cancer cell line. TBX3 was found to be one of HOXB13 protein partners. Both proteins, HOXB13 and TBX3, are required for the growth and proliferation of the same prostate cancer cell lines. Thus, HOXB13 and TBX3 are potential targets for developing new inhibitors for the treatment of prostate cancer

## Materials and methods

Immunoprecipitation. Immunoaffinity purification experiments were performed as previously described (Chetverina et al., 2022). The nuclear extract was obtained from the PC-3 cell line. 109 cells were washed twice in ice-cold PBS and resuspended in 10 mL of ice-cold Sucrose buffer (10 mM Tris, pH 7.5; 10 mM NaCl, 10 mM MgCl2, 1 mM EDTA, 1 mM EGTA, 1 mM DTT, 250 mM sucrose, EDTA-free protease inhibitor cocktail). Cells were homogenized using a Dounce pestle and incubated on ice for 10 min. The nuclei were then pelleted by centrifugation at 3,000g, +4 °C for 10 min. The pellet was resuspended in 1 mL of IP-500 buffer (10 mM Tris, pH 7.5; 500 mM NaCl, 10 mM MgCl2, 1 mM EDTA, 1 mM EGTA, 1 mM DTT, 0.1 % NP-40, 10 % glycerol, EDTA-free protease inhibitor cocktail), homogenized with a Dounce pestle and incubated for 1h at +4 °C on a rotator. Lysates were cleared by centrifugation at 18,000g, +4 °C for 10 min. The nuclear extract was then diluted to a final NaCl concentration of 150 mM using IP-0 buffer (10 mM Tris, pH 7.5; 10 mM MgCl2, 1 mM EDTA, 1 mM EGTA, 1 mM DTT, 0.1 % NP-40, 10 % glycerol, EDTA-free protease inhibitor cocktail).

Monoclonal antibodies against HOXB13 (EPR17371, ab201682, Abcam) or the IgG of non-immunized rabbits used as a negative control (Jackson ImmunoResearch #011-000- 002) were covalently coupled to the protein A Sepharose beads (Pierce) using DMP (Sigma). The nuclear extract containing 150 mM NaCl was incubated with antibodies and Sepharose for 14 h at +4 °C. After washing procedures, the resulting immunoprecipitates were eluted with buffer containing 2 % SDS, 100 mM Tris pH 8.0, 0.5 mM EDTA. Next, the probes were precipitated by TCA, followed by liquid chromatography/ tandem mass spectrometry (LC-MS) procedures

Mass spectrometry analysis of samples. The obtained samples were analyzed as previously described (Chetverina et al., 2022). Sodium deoxycholate (SDC) reduction and alkylation buffer, pH 8.5 (20 μL), containing 100 mM Tris, 1 % (w/v) SDC, 10 mM TCEP and 20 mM 2-chloroacetamide, was added to a 20-μg of each protein sample. Each sample was sonicated in an ultrasonic water bath for 1 min, heated at 95 °C for 10 min, cooled to a room temperature, and an equal volume of trypsin solution in 100 mM Tris pH 8.5 was added in a 1:50 (w/w) ratio. After overnight digestion at 37 °C, peptides were acidified by 40 μL of 2 % trifluoroacetic acid (TFA), mixed with 80 μL of ethyl acetate and purified using SDB-RPS StageTips. After washing the StageTips with 1 % TFA/ethyl acetate 1:1 mixture (2 times) and 0.2 % TFA (1 time), peptides were eluted into a clean tube by 50 % acetonitrile/5 % ammonia mixture. The collected material was vacuum-dried and stored at –80 °C. Before analyses, the peptides were dissolved in 2 % acetonitrile/0.1 % TFA buffer and sonicated for 1 min

The raw data and detailed protocol of the liquid chromatography and mass spectrometry experiments are publicly available in PRIDE (http://www.ebi.ac.uk/pride), project number PXD059115. The top 20 nuclear proteins were selected according to the following parameter: HOXB13 Spectral count/IgG Spectral count>=2. The TNMplot resource was used for GO analysis (Bartha, Győrffy, 2021).

Analysis of the sensitivity of cancer cell lines and gene expression levels in clinical samples. The analysis was based on the DepMap database (https://depmap.org/portal/). The CRISPR release (DepMap Public 24Q4+Score, Chronos) was used to analyze the data obtained by the CRISPR method. The RNAi release (Achilles+DRIVE+Marcotte, DEMETER2) was used to analyze the data obtained by the RNAi method.

Gene expression in tumor samples and the respective normal tissues was evaluated with the Mann–Whitney test using the TNMplot database (https://tnmplot.com), which contains transcriptome data from The Cancer Genome Atlas (TCGA) and The Genotype-Tissue Expression (GTEx) repositories (Bartha, Győrffy, 2021).

The survival analysis was carried out using the UCSC Xena database (http://xena.ucsc.edu/) (Goldman et al., 2020), using TCGA Prostate Cancer (PRAD) cohort, Illumina HiSeq 2000 RNA (dataset ID – TCGA.PRAD.sampleMap/HiSeqV2) and “Primary tumor” filter.

## Results

**The HOXB13 protein interactome in the PC-3 prostate cancer cell line**

**Fig. 1.**
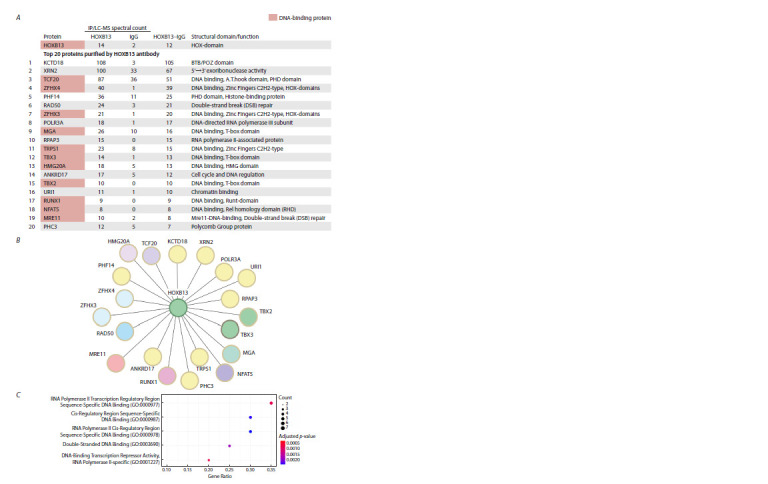
Transcription factors of the HOXB13 interactome in PC-3 prostate cancer cells A – spectral counts of the 20 nuclear proteins displaying the highest enrichment in the IP/LC-MS analysis (top 20). The HOXB13 and IgG columns show the results of the IP/LC-MS analysis performed using HOXB13-specific antibodies or IgG from a non-immunized animal, respectively. The HOXB13–IgG column shows the difference between the HOXB13 and IgG signals. The type of DNA-binding domain is indicated in the rightmost column. B – schematic representation of the HOXB13 interactome. C – GO analysis of the top 20 nuclear proteins enriched in the IP/LC-MS analysis

The HOXB13 protein partners were identified using immunoaffinity purification (IP) followed by liquid chromatography/ tandem mass spectrometry (IP/LC-MS) analysis. For this, we used the PC-3 prostate cancer line, which shows a high level of HOXB13 gene expression. A nuclear extract was isolated from the PC-3 cell line and incubated with either antibodies against HOXB13 or IgG from a non-immunized animal (the negative control), both of which had been coupled to Protein A Sepharose. After immunoprecipitation and a series of washes, the proteins were eluted from the Sepharose beads using SDScontaining buffer and analyzed by LC-MS mass spectrometry. Figure 1A, B shows 20 proteins with the highest enrichment in the IP/LC-MS analysis.

GO analysis of 20 nuclear proteins with the highest signal enrichment in the mass spectrometry analysis revealed that 11 out of 20 were DNA-binding transcription factors (Fig. 1C).

The two homologous proteins ZFHX4 and ZFHX3 (zinc finger homeobox 4 and 3, respectively) each contain 17 nonclustered C2H2-type zinc finger motifs and four homeoboxtype DNA-binding domains (HOX domains) (Fig. 1A). The TRPS1 protein also contains several non-clustered C2H2-type zinc finger motifs. The HMG20A protein contains an HMG (high mobility group) DNA-binding domain. Three factors have a T-box-type DNA-binding domain: the MGA (MAX gene-associated protein) and two homologue proteins TBX3 and TBX2 (T-box transcription factors 3 and 2, respectively). The TCF20 protein has an A.T.hook domain; the RUNX1 protein has a Runt domain; the NFAT5 protein has an RHD domain; and the MRE11 protein has a Mre11 domain (Fig. 1A).

Thus, many of the top HOXB13 protein partners are transcription factors that have different types of DNA-binding domains.

**DepMap database analysis: proteins encoded by the HOXB13 and TBX3 genes are most significant for proliferation of prostate cancer cell lines**

Next, to determine the functional significance of the identified HOXB13 partners, we queried the DepMap database to find out which cancer types are most affected by the knockout (CRISPR) or knockdown (RNAi) of genes encoding HOXB13 or its protein partners (see the Table). The DepMap project contains data from the screening of a large panel of cancer cell lines originating from various tissues. The project uses CRISPR or RNAi methods to investigate the dependence of cell proliferation on the suppression of individual genes (Tsherniak et al., 2017; Vazquez, Sellers, 2021). The probability of each cell line being dependent on the queried gene is represented by the “Gene effect” score, where strong negative values indicate that a given gene is particularly important for the growth and survival of the respective cell line.

**Table 1.**
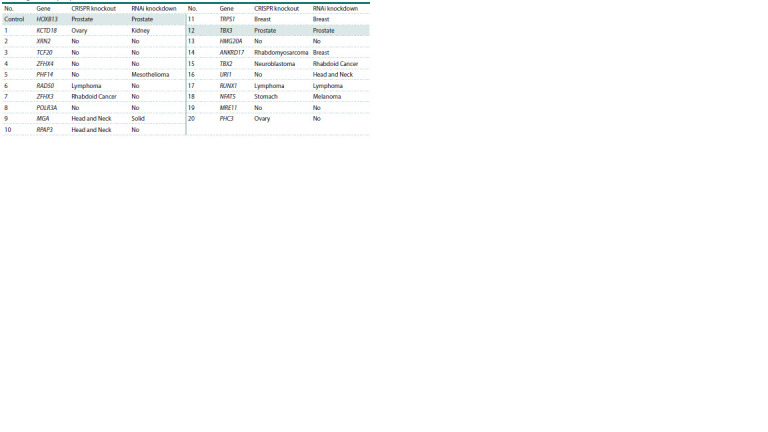
The tumor types that are most sensitive to depletion of HOXB13 or its partners by CRISPR knockout or RNAi knockdown, according to the DepMap database Note. A “No” designation indicates that none of the tumor types displayed preferential sensitivity to the inactivation of a given gene.

**Fig. 2.**
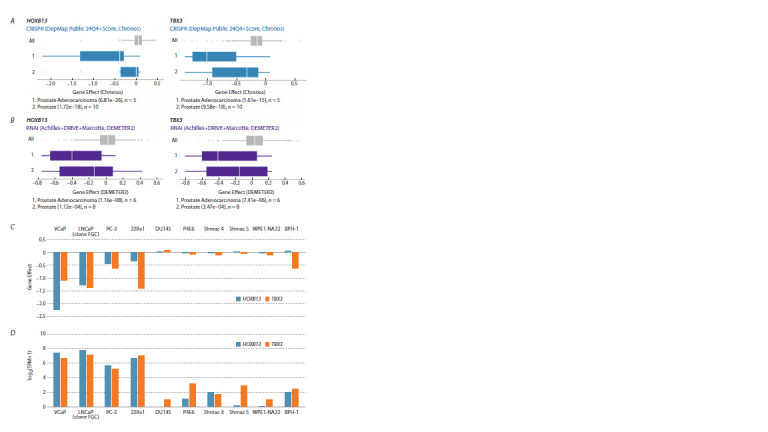
Prostate cancer cell lines are the most sensitive to knockout and knockdown of the HOXB13 and TBX3 genes. The analysis was performed using the DepMap database. A – DepMap analysis revealed that the knockout (CRISPR) of the HOXB13 (left) or TBX3 genes (right) specifically affects the growth and survival of cell lines of prostate origin. The effects of the test gene deletion on all cell lines are greyed out (1,178 cell lines in total). The effects of the test gene deletion on the cell lines of prostate origin are shown in blue. The X-axis shows the Gene Effect scores, which reflect the level of cancer cell proliferation when the tested gene is deleted. The lower the Gene Effect score, the more negative the effect of inhibiting gene activity on cell growth. The median value of the Gene Effect score is indicated by a white space. The analysis of DepMap reveals two groups of lines that are most specifically sensitive to knockout: 1 – the average effect on five lines corresponding to prostate adenocarcinoma; 2 – the average effect on ten prostate-origin cell lines, including those from adenocarcinoma. B – knockdown (RNAi, purple) affects the growth and survival of prostate-origin cell lines. The two groups of cell lines that are most specifically sensitive to knockdown are: 1 – the average effect on six prostate adenocarcinoma cell lines; 2 – the average effect on eight cell lines originating from the prostate, including adenocarcinoma cell lines. C – the effect of the HOXB13 or TBX3 genes knockout using CRISPR on the inhibition of proliferation of ten prostate cell lines, analyzed individually. The Gene Effect scores are indicated on the Y-axis. D – the Y-axis shows the transcription levels (log2(TPM+1)) in ten cell lines originating from prostate tissue. The expression data shown are for cell lines without gene knockout.

Analysis of the DepMap data revealed that tumor cell lines of different tissue origins respond differently to the depletion of the tested genes. Notably, disruption of either the TBX3 or HOXB13 genes activity leads to the preferential death of prostate cancer cell lines. Furthermore, impaired growth and proliferation of prostate tumor cells was observed for both HOXB13 and TBX3 using two depletion methods: knockout (CRISPR) and knockdown (RNAi) (Fig. 2A, B, see the Table).

To understand which cell lines are most sensitive to HOXB13 and TBX3 genes deletions, the cell lines were analyzed individually (Fig. 2C). The DepMap resource contains data on the effects of CRISPR knockout in 10 lines originating from prostate tissue. Five of them (VCaP, LNCaP (clone FGC), PC-3, 22Rv1 and DU145) originated from aggressive adenocarcinomas. The P4E6, Shmac 4, Shmac 5 lines are derived from cells of well or moderately differentiated nonmetastatic prostate carcinomas additionally immortalized by expression of the E6 HPV gene (Lang et al., 2006). WPE1- NA22 was derived from the normal prostate RWPE-1 cells following exposure to a chemical carcinogen (MNU) (Webber et al., 2001). BPH-1 was established from primary prostate epithelial cells by immortalization with SV40 large virus T antigen (Hayward et al., 1995).

Four out of five prostate adenocarcinoma cell lines were highly sensitive to the deletion of both the HOXB13 and TBX3 genes: VCaP, LNCaP (clone FGC), PC-3 and 22Rv1. By contrast, the deletion of HOXB13 and TBX3 was not significant for growth and proliferation of the DU145, WPE1-NA22, P4E6, Shmac 4, and Shmac 5 cell lines. The BPH-1 cell line was sensitive to the deletion of TBX3, but not HOXB13. Analysis of HOXB13 and TBX3 genes transcription in the studied lines (data from the DepMap resource) revealed that these factors exhibited the highest level of transcription in the VCaP, LNCaP (clone FGC), PC-3 and 22Rv1 lines, which are sensitive to the knockout of both genes (Fig. 2D). Thus, HOXB13 and TBX3 are preferentially required for the proliferation of cell lines originating from prostate adenocarcinoma samples.

**Fig. 3.**
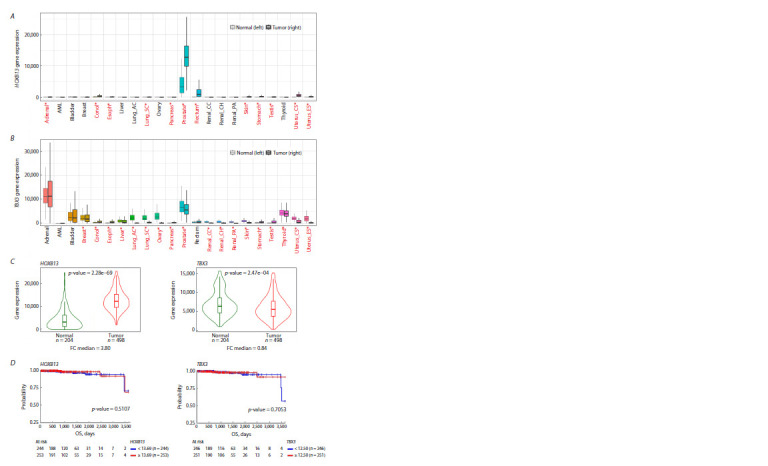
Expression of the HOXB13 and TBX3 genes in clinical samples. A, B – differential HOXB13 and TBX3 gene expression in normal (left) and tumor (right) samples of different tissues, analysis performed using TNMplot (Pan-cancer box plot). Cases with p-value < 0.05 (Mann–Whitney test) and expression >10 in tumor or normal samples are marked in red. С – the differential HOXB13 and TBX3 gene expression in normal (green) vs. prostate adenocarcinoma tumor (red) samples is presented as violin plots. p-value, Mann–Whitney test; FC – fold change median, analysis performed using TNMplot. D – correlations between HOXB13 and TBX3 gene transcription levels in prostate adenocarcinoma clinical samples and the overall survival (OS); analysis performed using the UCSC Xena resource. Cohorts with high gene expression are highlighted in red; cohorts with low expression are highlighted in blue.

Next, we analyzed the transcription levels of the HOXB13 and TBX3 genes in clinical samples using the TNMplot resource. HOXB13 transcription is normally restricted to prostate tissues (Fig. 3A). In clinical tumor samples, high levels of HOXB13 gene transcripts are observed in prostate and rectal tumor tissues. The TBX3 gene is transcribed in a greater number of tissues, with the highest expression levels being observed in the adrenal, prostate and thyroid samples (Fig. 3B). The levels of TBX3 transcription are often lower in tumor tissues than in normal samples, including those from prostate cancer. Figure 3C details the changes in the expression levels of the HOXB13 and TBX3 genes in normal and tumor prostate tissues. The HOXB13 gene showed a significant increase in transcription levels (FC = 3.8, p-value = 2.28e-69), while TBX3 transcription levels showed a slight decrease (FC = 0.84, p-value = 2.47e-04). However, no significant correlation was found between HOXB13 and TBX3 transcription levels and the overall survival of patients diagnosed with prostate adenocarcinoma (Fig. 3D).

## Discussion

In the present study we used IP/LC-MS to identify the protein partners of HOXB13 in the PC-3 prostate cancer cell line. We show that many of the HOXB13 partners are DNA-binding proteins.

One of the discovered partners of HOXB13 is the TBX3 protein, which has a T-box type DNA-binding domain in its structure. DepMap portal data analysis revealed that knockout (CRISPR) and knockdown (RNAi) of both the HOXB13 and TBX3 genes most significantly inhibited the growth and proliferation of cell lines originating from prostate adenocarcinomas. Transcription levels of the HOXB13 gene, but not those of TBX3, are significantly higher in clinical samples obtained from patients with prostate adenocarcinoma compared to normal prostate tissue samples. However, no correlation was found between increased HOXB13 or TBX3 gene transcription levels and the overall survival of patients with prostate adenocarcinoma

It can be assumed that the HOXB13 and TBX3 proteins are closely related in terms of their function and that they participate in the same transcription regulation cascades. Potentially, the combined inhibition of HOXB13 and TBX3 activities may have a stronger inhibitory effect on prostate cancer cell proliferation than inactivation of these proteins individually. For the TBX3 protein, it has previously been shown that it can repress transcription of tumor suppressor genes such as p14ARF (Lingbeek et al., 2002; Yarosh et al., 2008). It is possible that the cooperation between HOXB13 and TBX3 could increase the repression of the transcription of a subset of tumor suppressors. Further testing is required to confirm this. The role of TBX3 has been investigated in liver and breast tumors (Khan et al., 2020), but to our knowledge, only one study has examined the function of this factor in prostate cancer cells (Hwang et al., 2022). Using the LNCaP cell line, J.H. Hwang et al. demonstrated the presence of various DNA-binding proteins, including TBX3, HOXB13, FOXA1, and AR, in the interactome of the transcriptional co-factor CREB5. Knockdown of TBX3 and FOXA1 reduced the viability of the LNCaP cells.

The two HOXB13 protein partners, ZFHX4 and ZFHX3, are homologues and each of them contains four homeobox domains. It is known that homeobox domains can form protein-protein interactions (Ortiz-Lombardia et al., 2017). It is possible that the HOXB13 and ZFHX4/ZFHX3 homeodomains can interact directly. This will need to be tested in the future.

Previous studies using the VCaP prostate cancer cell line have shown that HOXB13 precipitates with the EED protein, which is a component of the PRC2 repressor complex (Cao et al., 2014). The study also revealed effective EED interaction with the PRC1 Polycomb repressor complex. In the current study, we did not detect any PRC2 complex subunits in the HOXB13 immunoprecipitate. However, two other Polycomb factors were identified: the PHC3 and RUNX1 proteins. PHC3, like its homologues PHC1 and PHC2, is the core subunit of the PRC1 subcomplex, which is known as cPRC1 (Schuettengruber et al., 2017). The transcription factor RUNX1 was previously shown to interact with the PRC1 core component BMI1 (a.k.a. PCGF4) (Yu et al., 2012). HOXB13 may potentially be involved in the regulation of transcription together with the Polycomb group of repressors. Further studies are required to investigate this.

To date, considerable attention has been devoted to identifying new targets for cancer therapy, with transcription factors being among the most promising ones (Bouhlel et al., 2015; Hagenbuchner, Ausserlechner, 2016; Lambert et al., 2018). There is growing evidence that suggests a key role for DNA-binding transcription factors in the processes of malignant tumor development (Vishnoi et al., 2020; Zhang et al., 2020). For example, analysis of the DepMap database identifies transcription factors as a crucial class of genes, the expression of which is critical for tumor cell proliferation (Chetverina et al., 2023). To date, methods have been developed to inhibit the activity of DNA-binding transcription factors. This makes transcription factors relevant subjects for study as potential targets for cancer treatment (Bushweller, 2019; Li et al., 2022; Zhuang et al., 2022; Xie et al., 2023).

The results of this study show that a large proportion of the top HOXB13 interactome proteins are DNA-binding factors. The ability of transcription factors to interact with other DNA-binding proteins is probably one of the most common mechanisms for regulating gene transcription in mammals (Jolma et al., 2013, 2015) and other multicellular organisms (Erokhin et al., 2018). Apparently, transcription factors can form ordered macro-complexes through multiple interactions, allowing them to recognize not only single, often degenerate binding sites, but also more extended regions of DNA consisting of a set of motifs for several proteins. The ability of DNA-binding proteins to interact with each other may be crucial for the specific selection of the DNA regions for the binding of regulatory complexes. Modulating the ability of individual DNA-binding proteins to be recruited to chromatin could potentially enable more precise control of gene expression, making transcription factors a promising target for the development of various anti-cancer drugs.

## Conclusion

The following main conclusions can be drawn based on the data obtained. 1. The main HOXB13 partners are transcription factors that have different types of DNA-binding domains. 2. Cell lines originating from prostate adenocarcinoma samples are most sensitive to deletion of the HOXB13 and TBX3 genes. 3. Suppression of HOXB13 and TBX3 gene activities inhibits the growth and proliferation of the same prostate adenocarcinoma cell lines. Further studies are required to understand the effects of co-inhibition of HOXB13 and TBX3 in vitro and in vivo

## Conflict of interest

The authors declare no conflict of interest.
